# Economic stress and child outcomes: the family stress model among Asian American families during COVID-19

**DOI:** 10.3389/fpsyg.2025.1591730

**Published:** 2025-07-30

**Authors:** Annie Fanta, Sei Eun Kim, Cindy J. Huang, William Tsai, Cindy Y. Huang

**Affiliations:** ^1^Department of Counseling and Clinical Psychology, Teachers College, Columbia University, New York, NY, United States; ^2^Department of Family Science and Human Development, Montclair State University, Montclair, NJ, United States; ^3^Steinhardt School of Culture, Education, and Human Development, New York University, New York, NY, United States; ^4^Prevention Science Institute, University of Oregon, Eugene, OR, United States

**Keywords:** economic stress, positive parenting, Asian American families, parent psychological distress, child internalizing and externalizing behavioral problems

## Abstract

Limited research has examined the impact of financial stress on families since the onset of COVID-19, specifically among Asian American (AA) parents. Informed by the Family Stress Model, this study examined the short-term longitudinal links between economic stress, parental psychological distress (i.e., anxiety and depression symptoms), positive parenting behaviors, and child outcomes three months later (i.e., child internalizing and externalizing problems). Results indicated that Time 1 (T1) parental economic stress was directly and positively associated with positive parenting behaviors at T1 and child internalizing problems at Time 2 (T2). Parental psychological distress at T1 was directly and negatively associated with positive parenting behaviors at T1. Positive parenting behaviors at T1 were directly and negatively associated with child externalizing problems at T2. Parental economic stress at T1 had an indirect effect on child externalizing problems at T2 through parenting behaviors. These findings highlight the need for clinical interventions targeted at improving parent mental health and promoting positive parenting behaviors during times of economic hardship to prevent adverse child outcomes. Furthermore, policy efforts aimed at alleviating economic stress among at-risk families can help reduce social inequality and buffer the harmful effects of economic stress on AA parents and families.

## Introduction

The onset of the COVID-19 pandemic posed a variety of stressors for families across the United States (U.S.), including an increase in financial insecurity (Prime et al., 2020). Particularly vulnerable to increased economic stress were low-income families, as they were more likely to hold jobs in sectors that were most severely impacted by the pandemic (Cubrich, 2020). With Asian American (AA) individuals overrepresented in these high layoff sectors (i.e., food services, retail, rideshare, beauty services), AAs experienced the sharpest increase in unemployment rates compared to other racial/ethnic groups during the early months of the pandemic (Kormendi and Brown, 2021). From February to May 2020, the national unemployment rate for AAs increased from 2.5 to 15% (Khan and Shih, 2021). Furthermore, rates of anti-Asian discrimination surged as AAs were perceived as COVID-19 carriers and blamed for the onset of the pandemic, resulting in increased hate crimes against AA individuals that included physical assaults and business vandalization (Tessler et al., 2020). This rise in anti-Asian racism also led to an avoidance of AA businesses with restaurants in Chinese neighborhoods closing at higher rates than food businesses in other neighborhoods (McGarity-Palmer et al., 2024). When the first reports of COVID-19 surfaced, the sales of some businesses in New York City’s Chinatown dropped 85% well before the first stay-at-home mandates (Roberts, 2020). In Southern California, two in five business owners reported that anti-Asian racism impacted their business through various forms such as vandalism, assault, and harassment (Ong et al., 2021). From the same sample, one in six businesses reported changing their operations due to safety concerns related to racism and a tenth of businesses reported a decline in business specifically due to avoidance of AA people and businesses.

Economic stress is defined as the psychological response to economic hardship, such as negative financial events (i.e., job loss), low income, high debts compared to assets, and other factors that designate families as meeting the government threshold for poverty status (Neppl et al., 2016). For parents, an increase in economic stress is particularly concerning as it poses a threat to parental well-being and family functioning, which can then subsequently impact child outcomes. Given the disproportionate amount of job loss and financial insecurity experienced by AAs in the context of COVID-19, it is critical to examine how economic stress affected family functioning and well-being among AA families during this time period. Informed by the Family Stress Model (FSM) (Conger and Conger, 2002), this study examined the links between economic stress, parental psychological distress (i.e., anxiety, depression), positive parenting behaviors, and child outcomes among a sample of AA families during the COVID-19 pandemic.

### Economic stress and family well-being

The adverse effects of economic stress on family well-being are clearly established in the extant literature. Among the most prominent outcomes associated with economic stress are parental psychological distress and harsh parenting, which can negatively impact a child’s social and emotional adjustment (Prime et al., 2020). These familial processes have been found to be consistent across a variety of racial/ethnic groups, family structures, and geographic locations (Masarik and Conger, 2017). Additionally, economic stress is a known risk factor for child maltreatment (Thor et al., 2022). Consistent with this, mothers experiencing employment and financial loss during the early months of the COVID-19 pandemic were more likely to score higher on a child abuse risk scale (Rodriguez et al., 2021).

Parental job loss during the COVID-19 pandemic, a key marker of economic stress, was associated with increases in parent–child conflict, which then predicted increases in child negative affect and decreases in child positive affect (Wang et al., 2021). Parental job and income loss were both found to predict parental stress, depressive symptoms, a reduced sense of hope, and negative parent–child interactions during the pandemic (Kalil et al., 2020). Conversely, job loss without income loss was not significantly associated with these adverse outcomes and instead predicted improved positive parent–child interactions (Kalil et al., 2020). These findings highlight how common markers of economic stress, such as job loss, impact families differently and may not necessarily lead to psychological stress over financial difficulties. For example, among high-income families with various financial safety nets (i.e., significant savings, dual-parent incomes, family wealth), job loss may result in less psychological distress compared to their low-income family counterparts.

### The family stress model

According to the FSM (Conger and Conger, 2002), economic stress indirectly affects child outcomes through disrupted family functioning, such as parental psychological distress, marital conflict, and negative parenting behaviors. According to this model, economic hardship is expected to lead to economic stress within the family and may include difficulties paying bills; food, clothing, and housing insecurity; and a lack of money for necessities such as medical care (Neppl et al., 2016). A parent who experiences economic stress is hypothesized to be at increased risk for psychological distress (i.e., anxiety and depressive symptoms), which, in turn, “spill over” into the marital relationship and parenting behaviors (Erel and Burman, 1995). Lastly, it is expected that marital conflict and negative parenting behaviors will adversely impact the social and emotional well-being of children.

A significant body of research has examined the various pathways in the FSM. First, the pathway between economic stress and parental psychological distress is supported by findings that show associations between economic stress and higher levels of parental psychological distress, such as anxiety, depression, hopelessness, hostility, and feelings of discouragement (Landers-Potts et al., 2015; Newland et al., 2013). When parents experience psychological distress, they often become easily upset, found it difficult to relax, and felt a limited ability to successfully navigate daily stressors (Low and Mounts, 2022).

The next pathway in the model suggests that parental psychological distress leads to marital problems and disrupted parenting. Across a variety of populations, parental psychological distress that is directly associated with economic stress has been found to also be associated with marital conflict, less partner support, negative perceptions of marriage, and lower relationship satisfaction (Helms et al., 2014; Landers-Potts et al., 2015; Neppl et al., 2016). Furthermore, a significant body of research indicates that parental psychological distress is associated with higher levels of harsh parenting (Neppl et al., 2016), more parent–child relationship conflict (Russell et al., 2020), less supportive and sensitive parenting (Newland et al., 2013). Finally, the third FSM pathway proposes a relationship between negative parenting and child outcomes and is supported by research that links negative and hostile parenting to increased child externalizing (i.e., conduct problems and hyperactivity/inattention difficulties) and internalizing behaviors (i.e., emotional and relationship problems) (Masarik and Conger, 2017).

While many FSM studies focus on harsh or negative parenting, it is also important to consider how positive parenting behaviors fit into the model. Given the negative association between parent psychological distress and positive parenting behaviors (Ludmer et al., 2017), this is a critical gap in the FSM literature. Existing research describes positive parenting behaviors as proactive parenting (e.g., giving clear choices), parental warmth (e.g., providing opportunities for open communication between parent and child), quality time (e.g., doing enjoyable activities together), limit setting (e.g., setting clear and consistent expectations), and positive reinforcement (e.g., praising the child; Dishion et al., 2008). These positive parenting behaviors contribute to a variety of adaptive child outcomes, such as warm and trusting relationships, attentive involvement, self-regulation (Horner and Carr, 1997; Dishion et al., 2008), prosocial behaviors (Nelson et al., 2009), lower levels of conduct and behavior problems (Gardner et al., 2007), academic success (Sorkhabi and Middaugh, 2019), and less substance use (Kosterman et al., 2000). In addition to cross-sectional studies, longitudinal research has further supported the association between positive parenting and lower internalizing (Yap et al., 2016) and externalizing behaviors (Boeldt et al., 2012) later in childhood and adolescence.

Despite substantial empirical support for the FSM, there is limited research on the links between economic stress, family processes, and child outcomes among immigrant families (Mistry et al., 2009). Specifically, the majority of research on the FSM focuses on European American and African American populations (Davis et al., 2020), with few studies testing the validity of the model among AA families. With the sharp rise in anti-Asian discrimination and hate crimes at the onset of the pandemic, AA parents were at increased risk for psychological distress and economic vulnerability (Cheah et al., 2020). Thus, the spillover effects of parental psychological distress into family processes such as parenting behaviors and subsequent child outcomes may be particularly prominent for AA families during this time. Given the influence that Asian cultural values and beliefs (i.e., collectivism, emotional self-control, humility, and conformity to norms) can have on family processes such as parenting behaviors (Park et al., 2010), in addition to the increased stressors associated with the COVID-19 pandemic, it is necessary to understand if the FSM can be generalized to AA families or if adaptations are needed to more appropriately capture AA family processes stemming from economic stress.

## Current study

To date, no studies have examined the FSM among AA families within the context of the COVID-19 pandemic, a critical time to understand how economic stress may have directly and indirectly impacted the well-being of AA parents and children. Thus, the current study examined the short-term longitudinal relations between (1) AA parents’ economic stress, (2) parental psychological distress (i.e., anxiety and depressive symptoms), and (3) positive parenting behaviors (i.e., positive reinforcement, proactive parenting, parenting warmth, limit setting, quality time, and negative parenting) at baseline and child externalizing and internalizing problems 3 months later. For this study, externalizing behaviors refer to conduct problems and hyperactivity/inattention difficulties while internalizing behaviors are defined as emotional and relationship problems.

It is important to note that the term “parenting behaviors” in this study refers specifically to *positive* parenting behaviors, which include high levels of positive reinforcement, proactive parenting, parenting warmth, limit setting, and quality time, as well as low levels of negative parenting. The focus on positive parenting behaviors is unique compared to many previous FSM studies, which examine the use of negative or harsh parenting behaviors (Masarik and Conger, 2017). By specifically focusing on positive parenting behaviors, this study aims to explore how parents can potentially *protect* children from the risks associated with exposure to economic stress. Additionally, despite the significant body of research supporting the FSM, many studies utilize common markers of economic stress (i.e., low income) as proxies for the construct. However, using a proxy to measure economic stress may fail to capture the experiences of families who experience financial stress in other ways. For example, parents who have high or moderate incomes may still experience psychological stress associated with the threat of job loss. Therefore, it is important to note that this study utilized a measure of economic stress that is directly aimed at measuring the construct. However, it should be acknowledged that this measure of economic stress is not specific to COVID-19 and therefore economic stress that was the direct result of the pandemic was not captured, but rather economic stress during the pandemic was measured.

Based on previous literature, it was hypothesized that: (1) Economic stress will be positively associated with parental psychological distress; (2) parental psychological distress will be negatively associated with positive parenting behaviors; (3) positive parenting behaviors will be negatively associated with child externalizing and internalizing behaviors at 3-month follow-up; (4) economic stress will be indirectly associated with positive parenting behaviors through parental psychological distress; (5) parental psychological distress will be indirectly associated with child externalizing and internalizing behaviors at 3-month follow-up through positive parenting behaviors.

## Method

### Procedure

This study utilized data from a longitudinal study examining the effects of the COVID-19 pandemic on the mental health of AA parents and youth. Participants were recruited across two cohorts through social media (e.g., Instagram and Facebook) and professional listservs (e.g., Asian American Psychological Association Listserv) as well as in-person at a community health center in New York City. Parents were approached by members of the research team in the pediatric department of the health center. Both in-person and online recruitment efforts were targeted towards all individuals who self-identified as Asian American and living in the U. S. If interested in participating, participants were provided the online survey which could be completed in English or Chinese. Individuals who met the following criteria were eligible for the study: (a) identified as Asian American, (b) the parent of a child between 2 and 19 years old in school (excluding college) and living at home, and (c) currently residing in the U.S. If eligible, participants completed a 45-min Qualtrics survey online which consisted of self-report measures about their feelings regarding the COVID-19 pandemic, their parenting behaviors, and their child’s behaviors. Surveys were age-normed for three different age groups: early childhood (2–5 years), middle childhood (6–10 years), and adolescents (11–19 years). If participants had more than one child, they were instructed to choose one child when completing the survey. Participants were followed up at 3-months (T2), 6-months (T3), and one year (T4). The current study utilized data from baseline (T1) and 3-month follow-up (T2). Participants received a $10 Amazon gift card for each survey they completed. This study was approved by the [*blinded for review*] Institutional Review Board.

### Participants

The study sample included 282 AA parents (*M_age_* = 40.6 years, *SD* = 7.43). The majority of parents were female (80.1%), married (89.1%), and had a college or graduate degree (74.3%). Sixty-three percent of the parents were foreign-born (i.e., first-generation immigrants) and 36.2% were U. S.-born. Fifty-eight percent of the participants resided in suburban areas, followed by 37.7% in urban areas and 3.4% in rural areas. Parents reported on children who averaged 11.59 years old (*SD* = 7.84) and were 52.8% female and 47.2% male. The sample was representative of a diverse number of Asian subgroups, with 52.7% identifying as Chinese, 24.4% as Taiwanese, 4.6% as Korean, 2.1% as Filipino, 2.1% as Japanese, 1.8% as Vietnamese, 0.7% as Indonesian, 0.4% as Hmong, and 8.2% as multi-ethnic. Geographically, the majority of the participants were from the Northeast (54.6%), followed by the West (33.5%), South (7.4%), and Midwest (4.5%). Additional demographic information can be found in Table 1.

**Table 1:** Descriptive of demographic characteristics.

Variables	Frequency (%)
Gender	
Male	56 (19.9%)
Female	226 (80.1%)
Marital status	
Married	246 (89.1%)
Living together	5 (1.8%)
Separated	6 (2.2%)
Divorced	8 (2.9%)
Single	2 (7%)
Missing	15
Ethnicity	
Chinese	149 (52.7%)
Taiwanese	69 (24.4%)
Korean	13 (4.6%)
Filipino	6 (2.1%)
Vietnamese	5 (1.8%)
Japanese	6 (2.1%)
Hmong	1 (0.4%)
Indonesian	2 (0.7%)
Multi-ethnic	13 (8.2%)
Missing	18
Neighborhood	
Urban	126 (45.7%)
Suburban	141 (51.1%)
Rural	8 (2.9%)
Other	1 (0.4%)
Missing	
Education attainment	
High school degree or less	50 (14.5%)
Partial college training	21 (7.6%)
College degree	87 (31.5%)
Graduate degree	118 (42.8%)
Missing	6

	M (SD)
Age	40.6 (7.43)

### Measures

#### Economic stress (T1)

Economic stress was measured using five items from the Economic Stress Index (ESI) (Kling et al., 2007). The measure included items such as, “How often do you worry about being able to meet your monthly living expenses?,” “Would you say you worry all the time, very frequently, occasionally, rarely, very rarely, or never?,” and “In the past 12 months, would you say that your household has spent more, less or about as much as all of your sources of income combined?” The composite scores of all 5 items were calculated by taking the sum of the items (Troller-Renfree et al., 2022). Higher scores indicated greater levels of economic stress. This measure demonstrated an acceptable internal consistency reliability in this study (Cronbach’s *α* = 0.70) and has reliably been used with a diverse sample of parents (Troller-Renfree et al., 2022).

#### Psychological distress (T1)

Psychological distress was assessed by the Depression and Anxiety subscales of the Depression and Anxiety Stress Scales (DASS-21) (Lovibond and Lovibond, 1995). Both subscales included seven items that ask about participants’ feelings in the past week on a 4-point Likert scale (*0-Never* to *3-Almost Always*). Higher scores indicated higher levels of depressive and anxiety symptoms. Examples of items on the depression subscale include: “I was unable to become enthusiastic about anything” and “I could not seem to experience any positive feeling at all.” The anxiety subscale consists of items such as: “I felt scared without any good reason” and “I was worried about situations in which I might panic and make a fool of myself.” The Depression and Anxiety subscales were combined to create the psychological distress variable, which demonstrated good internal consistency in this study (Cronbach’s α = 0.91). Additionally, the DASS-21 has shown good concurrent validity with other anxiety and depressive measures (Osman et al., 2012) and good validity among Asian samples (Norton, 2007).

#### Parenting behaviors (T1)

Six measures were used to assess parenting behaviors, which included proactive parenting, quality time, limit setting, positive reinforcement, parental warmth, and negative parenting. Total scores for each subscale were calculated as averages. Higher scores on each of the parenting behavior measures indicated a greater likelihood of the parent exhibiting the respective parenting behavior. All parenting measures have been validated with diverse samples (McEachern et al., 2012; Ringle et al., 2019).

##### Proactive parenting

Proactive parenting can be defined as a parent’s ability to anticipate potential problems and respond in a way that can help their child avoid engaging in problem behaviors (Dishion et al., 2008). Proactive parenting utilizes anticipatory skills rather than reactive strategies (Padilla-Walker et al., 2011). Proactive parenting was measured using seven items adapted from the Parenting Children and Adolescents (PARCA; unpublished instrument) instrument that assesses the frequency of proactive parenting behaviors, such as giving reasons for requests, breaking tasks into smaller tasks, and warning the child before a change of activity. The PARCA was adapted for use with adolescents from the Parenting Young Children measure (PARYC) (McEachern et al., 2012). Items were rated on a 5-point Likert scale (*0-Not at all* to *4-Very often*). In this study, the internal consistency reliability of this measure ranged from 0.75 to 0.80 across child age groups.

##### Quality time

Quality time is defined as a parent’s effort to spend quality time with their children and create opportunities for meaningful interactions such as engaging in shared activities and teaching children new skills (Dishion et al., 2008). Quality time was assessed using a five-item subscale of the Parenting Children and Adolescents (PARCA) instrument by evaluating behaviors such as spending time with the child, helping the child to learn something new, and involving the child in household activities (e.g., “How often do you play with your child in ways that were fun for both of you?”) The PARCA was adapted for use with adolescents from the Parenting Young Children measure (PARYC; McEachern et al., 2012). Items were rated on a 5-point Likert scale (*0-Never* to *4-Very Often*). In this study, the internal consistency reliability of this measure ranged from 0.76 to 0.81 across child age groups.

##### Limit setting

Limit setting is defined as setting clear rules and expectations and having consistent consequences (Dishion et al., 2008). Limit setting was measured using a seven-item subscale of the Parenting Children and Adolescents (PARCA) instrument by evaluating how often parents set expectations and rules on their child’s behavior (e.g., “How often do you tell your child how you expected him/her to behave?”) The PARCA was adapted for use with adolescents from the Parenting Young Children measure (PARYC) (McEachern et al., 2012). Items were rated on a 5-point Likert scale (*0-Never* to *4-Very Often*). In this study, the internal consistency reliability of this measure ranged from 0.79 to 0.85 across child age groups.

##### Positive reinforcement

Positive reinforcement is defined as a parent’s use of providing praise, physical affection, and providing tangible rewards that encourage positive and desired child behaviors (Dishion et al., 2008). Positive reinforcement was measured using adapted questions from the Parenting Children and Adolescents (PARCA, unpublished) instrument as well as the Community Action for Successful Youth (CASEY) (Metzler et al., 1998), which was developed and adapted from a National Institute on Drug Abuse-funded intervention trial through the Oregon Research Institute (ORI) (Biglan et al., 1994). Positive reinforcement was assessed using four items that asked parents to report how often they praised their child and rewarded their child for doing something well (e.g., “Reward your child when s/he did something well or practiced a new skill?”). Items were rated on a 5-point Likert scale (*0-Never* to *4-Very Often*). In this study, the internal consistency reliability of this measure ranged from 0.71 to 0.84 across child age groups.

##### Parental warmth

Parental warmth refers to the quality of the parent–child relationship and was measured using five items from the Adult-Child Relationship Scale (Criss and Shaw, 2005), which was adapted from the school-based Student-Teacher Relationship Scale (Pianta and Steinberg, 1991). This measure assessed a parent’s degree of warmth towards their child (e.g., “If upset, my child seeks comfort from me,” “My child likes telling me about him/herself,” and “It is easy to be in tune with what my child is feeling.”) Items were rated on a 5-point Likert scale (*1-Definitely Not* to *5-Definitely*). In this study, the internal consistency reliability of this measure ranged from 0.80 to 0.93 across child age groups.

##### Negative parenting

Negative parenting refers to the frequency of the parent having struggles in parenting their child and relying on harsh or critical strategies. This was measured using a five-item scale adapted from the unpublished instrument PAL-2 Caregiver Check-in, as part of the Child and Family Center Norm Study (Child and Family Center, 2005). Examples of items in this measure include “You criticize your child,” “You felt that you could not handle your child’s behavior,” and “You yelled or shouted at your child.” Items were rated on a 5-point Likert scale (*1-Not at all* to *5-Very Often*). In this study, the internal consistency reliability of this measure ranged from 0.65 to 0.74 across child age groups.

The six subscales were combined as a latent construct for parenting behaviors. The CFA indicated a good model fit: χ2(*df* = 2) = 18.81, *p* < 0.001; comparative fit index (CFI) = 0.95; root mean square error of approximation (RMSEA) = 0.07; standardized root mean square residual (SRMR) = 0.04.

### Child outcomes (T2)

Child outcomes at 3 months after baseline (T2) were assessed using the Strengths and Difficulties Questionnaire (SDQ) (Goodman, 1997). The parent-report measure is aged-normed and includes five subscales that measured emotional symptoms, conduct problems, hyperactivity-inattention, peer problems, and prosocial behavior. Each subscale consists of 5 items that use a 3-point Likert scale (*0-Never true* to *2-Certainly true*). For this study, the Peer Relationship Difficulties and Emotional Symptoms subscales were grouped together to create an Internalizing Problems subscale, and subscales for Conduct Problems and Hyperactivity were combined to create an Externalizing Problems subscale (Goodman et al., 2010). Higher scores indicated greater levels of the respective child outcome. The internalizing and externalizing subscales demonstrated good internal reliability (Cronbach’s *α* = 0.76 and 0.78, respectively).

### Covariates

Demographic characteristics that may be associated with the constructs of interest for this study were included in the analyses as covariates: parent-reported parent gender, parent age, parent education status, child gender, and state of residence (due to the potential impact of the length of state lockdown mandates on economic stress). Parent gender and child gender were dummy coded into two variables (*0-Male, 1-Female*). State of residence was dummy coded by geographic region (*0-Northeast, 1-Midwest, 2-West, 3-Southwest, 4-Southeast*), with Northeast set as the reference group.

### Data analysis

Descriptive analyses, reliability of constructs, and bivariate correlations were conducted using R statistical package (R Core Team, 2021). A path analysis using structural equation modeling (SEM) was conducted to test the direct and indirect effects of economic stress on study outcomes. Goodness-of-fit was determined based on chi-square tests (χ2), the comparative fit index (CFI), the root mean square error of approximation (RMSEA), and standardized root mean square residual (SRMR). A fit of > 0.95 for CFI, < 0.05 for RMSEA, and < 0.05 for SRMR is considered a good fit (Hu and Bentler, 1999). Modification indices (MI) function to test whether data suggested which model fit would improve if a particular path was added or constraint freed. Full information on maximum likelihood was used to compute the maximum likelihood estimates (Russell et al., 2020).

## Results

Bivariate correlation analyses were conducted to examine the relationships among all study variables. Economic stress was positively correlated with parental psychological distress at T1 (*r* = 0.32, *p* < 0.01), negative parenting at T1 (*r* = 0.16, *p* < 0.05), and child internalizing problems at T2 (*r* = 0.26, *p* < 0.01), but negatively correlated with parental warmth at T1 (*r* = −0.17, *p* < 0.01), quality time at T1 (*r* = −0.23, *p* < 0.01), positive reinforcement at T1 (*r* = −0.19, *p* < 0.01), proactive parenting at T1 (*r* = −0.21, *p* < 0.01), and limit setting at T1 (*r* = −0.21, *p* < 0.01). Parental psychological distress at T1 was positively correlated with negative parenting at T1 (*r* = 0.41, *p* < 0.01), child internalizing problems at T2 (*r* = 0.24, *p* < 0.01), and child externalizing problems at T2 (*r* = 0.21, *p* < 0.01), but negatively correlated with parental warmth at T1 (*r* = −0.22, *p* < 0.01), quality time at T1 (*r* = −0.31, *p* < 0.01), positive reinforcement at T1 (*r* = −0.20, *p* < 0.01), and limit setting at T1 (*r* = −0.21, *p* < 0.01). Detailed information on correlations can be found in Table 2.

**Table 2:** Means, standard deviations, and correlations for study variables.

Variable	*M*	*SD*	1	2	3	4	5	6	7	8	9	10	11
1. Age	40.65	7.43											
2. Gender	0.80	0.40	−0.13*										
3. Economic stress	2.93	0.95	−0.03	−0.06									
4. Psychological distress T1	1.49	0.47	−0.20**	−0.01	0.32**								
5. Parental warmth T1	4.12	0.81	−0.18**	0.19**	−0.17**	−0.22**							
6. Quality time T1	2.71	0.65	−0.22**	0.12	−0.23**	−0.31**	0.62**						
7. Positive reinforcement T1	3.14	0.80	−0.24**	0.11	−0.19**	−0.20**	0.56**	0.67**					
8. Proactive parenting T1	2.44	0.73	−0.22**	0.17**	−0.21**	−0.14*	0.49**	0.62**	0.60**				
9. Negative parenting T1	1.34	0.64	−0.02	0.08	0.16*	0.41**	−0.31**	−0.41**	−0.29**	−0.15*			
10. Limit setting T1	2.59	0.61	−0.11	0.03	−0.21**	−0.21**	0.42**	0.55**	0.51**	0.65**	−0.21**		
11. Internalizing problems T2	0.95	0.65	0.09	−0.07	0.26**	0.24**	−0.24**	−0.40**	−0.16*	−0.11	0.15	−0.17*	
12. Externalizing problems T2	1.15	0.72	0.02	0.09	0.15	0.24**	−0.10	−0.29**	0.03	0.06	0.37**	−0.05	0.48**

T1 refers to time-point 1; T2 refers to time-point 2. *indicates *p* < 0.05. **indicates *p* < 0.01.

**Figure 1:**
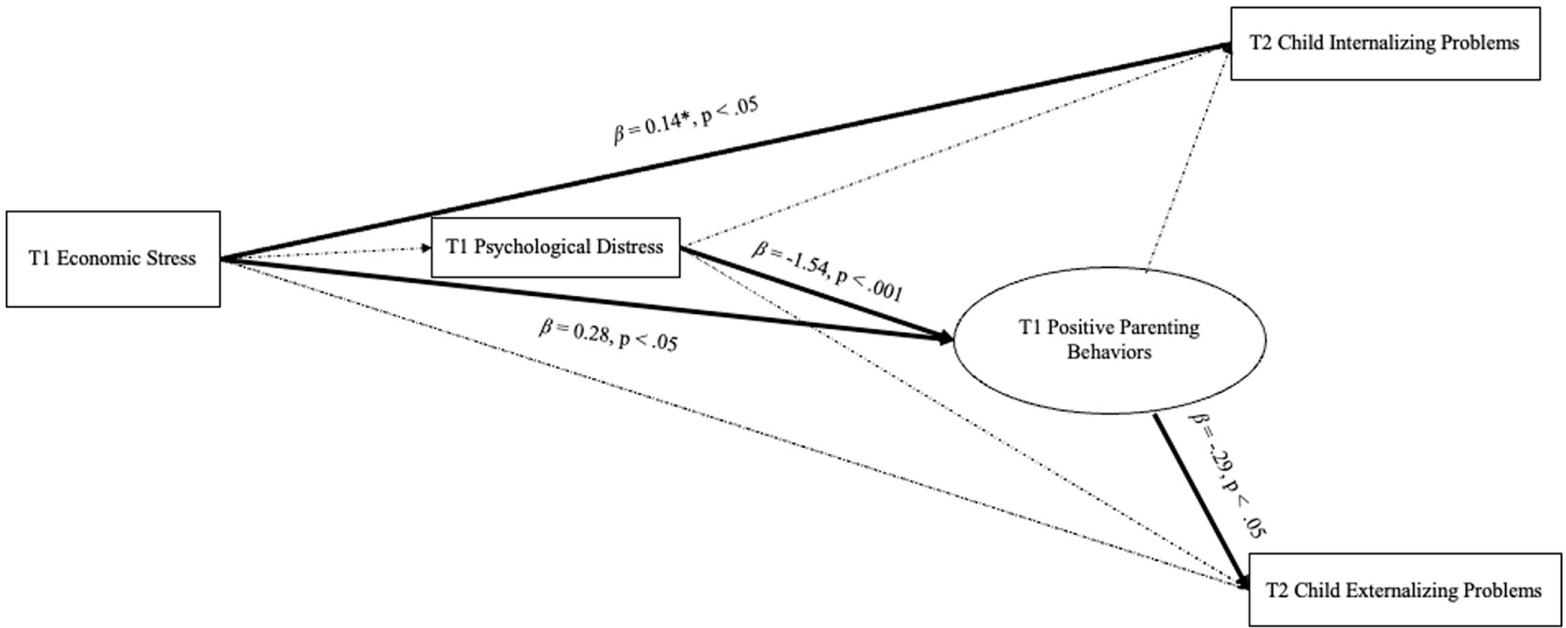
Path model examining economic stress at baseline (T1), parental psychological distress at baseline (T1), positive parenting behaviors at baseline (T1), and child externalizing and internalizing behaviors 3 months later (T2). Bolded paths indicate significance at *p* < 0.05.

The model fit indices suggested good model fit (χ2[Δdf = 31, *p* < 0.001, CFI = 0.94, RMSEA = 0.07, and SRMR = 0.05]). The path model demonstrated significant direct and indirect effects in the model which are displayed in Figure 1. First, T1 parental economic stress was directly and positively associated with positive parenting behaviors at T1 (*β* = 0.28, *p* < 0.05) and child internalizing problems at T2 (*β* = 0.14, *p* < 0.05). Parental psychological distress at T1 was directly and negatively associated with positive parenting behaviors at T1 (*β* = − 1.54, *p* < 0.001). Positive parenting behaviors at T1 were directly and negatively associated with child externalizing problems at T2 (*β* = −0.29, *p* < 0.05). Parental economic stress at T1 had an indirect effect on child externalizing problems at T2 through parenting behaviors (indirect effect point estimate = −0.08, *p* < 0.05). No other significant direct and indirect effects emerged.

## Discussion

Grounded in the FSM, this current study examined the direct and indirect relationships between economic stress, parental psychological distress, positive parenting behaviors, and child outcomes among AA families during the early months of the COVID-19 pandemic. Contrary to existing literature and study hypotheses, economic stress was not directly associated with parental psychological distress. Rather, economic stress led to increases in child internalizing problems over time, which highlights the unique impact of economic stress on AA families in the context of the COVID-19 pandemic. Most notable is the direct link between economic stress and child internalizing problems, which indicated that children whose parents experienced economic stress were at increased risk for internalizing problems 3 months later. This finding contributes to the FSM literature by demonstrating that economic stress can be felt and internalized by children directly, and that economic stress does not have to impact parent mental health or parenting behaviors for it to be harmful to the child.

Similarly, the direct link between economic stress and parenting behaviors suggests that economic stress can impact parenting regardless of the impact on parents’ psychological distress. The directionality of this relationship is important to note, as parents with higher levels of economic stress also reported higher levels of positive parenting behaviors. This finding is contradictory to the hypothesis of Conger and Elder (1995) and numerous studies showing longitudinal associations between financial hardship and harsh parenting (Conrad-Hiebner and Bryam, 2018). Thus, it is important to consider the context of the COVID-19 pandemic when interpreting this finding and the unique impact it may have had on family dynamics and parenting. For example, one possible explanation is that parents who experienced layoffs (i.e., a risk factor for economic stress), were able to spend more time at home, which subsequently allowed them to dedicate more energy to engaging in positive parenting behaviors. In fact, existing research found that AA parents and children spent an increased amount of quality time together during the early months of the pandemic due to school shutdowns (Huang and Tsai, 2023). This increase in quality time likely offered more opportunities for parents to engage in more proactive parenting, positive reinforcement, and limit setting with their children. With more time to engage in positive parenting strategies, parents may have found themselves using fewer negative parenting behaviors, such as yelling.

Another possible explanation for this finding is that parents might have felt more intentionality to protect their children from family economic stress during a time of crisis. Beyond just economic and employment risks, COVID-19 posed a variety of other stressors to families including health concerns, school closures, childcare needs, social isolation, and, for AA families specifically, increased risk of discrimination. Thus, parents may have viewed the home environment as one of the few domains in their life that they could control and where they could attempt to protect their children from the external stressors and uncertainty of the pandemic. As a result, parents may have channeled more energy into utilizing positive parenting behaviors to create a nurturing, predictable, and safe environment for their children.

Additionally, even parents who did not experience layoffs may have experienced stress around the *possibility* of losing their jobs and also spent more time at home due to the local, state, and national lockdown mandates and companies shifting to remote work. For example, a parent who worked in the restaurant industry may have retained their job throughout the lockdown but likely experienced concerns around the stability and long-term availability of their job. This parent may have also spent more time at home as opportunities for social and recreational activities were limited, which subsequently may have allowed them to engage in more positive parenting behaviors. While research is limited on positive parenting behaviors during the COVID-19 pandemic, a study of 572 low-income families found that 75% of parents in the sample reported spending more time caring for their young children during the COVID-19 pandemic, which was a significant predictor of positive parent–child interactions (Kalil et al., 2020) and corroborates the findings from this study. Furthermore, this potential explanation accounts for the resiliency and flexibility of AA parents who may have been able to implement or maintain adaptive parenting behaviors despite economic stress.

More consistent with past FSM literature, we found that greater levels of parental psychological distress were associated with lower levels of positive parenting. As prior research has posited, it is likely that when parents experience psychological distress, there may be “spillover” effects as the distress begins to negatively impact parenting behaviors and other family processes (Puff and Renk, 2014). Parents experiencing depressive symptoms may have less emotional and mental energy available to engage in positive parenting behaviors. Thus, the adverse effects of parental psychological distress can result in less-than-optimal parenting behaviors as evidenced by these results. Additionally, the protective effects of positive parenting behaviors were highlighted by the negative association between positive parenting behaviors and child externalizing problems. That is, regardless of how much economic stress a family was experiencing, when parents engaged in higher levels of positive parenting, children were less likely to demonstrate externalizing behaviors 3 months later. Given the increased mental health risk of AA youth during the pandemic, this finding is particularly important as it identifies specific parenting behaviors that AA parents were able to use to prevent children from developing externalizing problems.

Notable in the findings is the indirect relationship between economic stress and child externalizing behaviors through positive parenting behaviors. This suggests that even when parents experienced high levels of economic stress, the use of positive parenting behaviors may serve as a protective factor for children and their externalizing behaviors 3 months later. Put another way, what parents are doing can protect their children from the effects of economic stress by reducing the likelihood of children developing hyperactivity and conduct problems. Furthermore, this finding highlights the differential impact that economic stress can have on the development of child internalizing versus externalizing behaviors as the pathway between economic stress and child internalizing problems is occurring regardless of parent intervention.

One possible explanation for this finding is that when children witness their parents experiencing economic stress, they may internalize the stress themselves. Children who internalize this stress may then be more likely to develop feelings of worry or nervousness around familial finances, which could lead to the development of mental health problems Additionally, children who see their parents experiencing economic stress may begin to feel hopeless about the future of their family’s financial stability, which could develop into depressive symptoms. These explanations are supported by a study that found an association between parent-reported economic stress and Chinese American adolescents’ perception of family economic stress which, in turn, was linked to increased depressive symptoms among the adolescents (Mistry et al., 2009). Similarly, a study by Delgado et al. (2013) found that parent-report of economic stress was positively associated with adolescents perceived family economic stress which was indirectly related to adolescent depression symptoms among a sample of Mexican families.

Lastly, it is important to note the lack of a direct relationship between economic stress and psychological distress. When examining possible explanations for this, it is important to consider cultural factors that are specific to AA families. Traditional Asian cultural values, such as maintaining harmony, saving face, and family support, may influence how AA parents experience and manage economic stress. For example, an AA parent who is at risk for losing their job may conceal their psychological stress for fear of losing face and disrupting the family system. Given the shame that may be experienced, AA parents will likely aim to prevent the trickle-down effects of the economic stress on their children’s well-being and focus their efforts on protecting their children from experiencing adverse effects. These parents may also prioritize their children’s well-being over disclosing personal mental health struggles, aligning with face-saving norms and a collective orientation. To shield their children from the negative effects of economic hardship, AA parents may engage in positive parenting behaviors that preserve family stability. This active role likely provided a sense of control during the pandemic and helped buffer the impact of psychological distress, highlighting the protective function of collectivist values in times of adversity.

### Limitations and future directions

There are several limitations of this study worth noting. First, child outcomes were measured via parent self-report. While this was necessary given the age ranges of children included in the study, future research may benefit from using a multi-informant approach (e.g., child report, teacher report). Second, the sample of this study may not have captured the ethnic diversity of the AA population, as the majority of participants identified as Chinese and Taiwanese. Therefore, results may not generalize to other AA subgroups which is important to note. Future studies may benefit from recruiting a more diverse AA sample and examining differences in economic stress among AA subgroups given the disparities that exist in economic status among Asian ethnic groups. Specifically, the AA population has the highest economic gap compared to any other racial/ethnic group in the U.S. (Kochhar and Cilluffo, 2018). For example, although the median income for AA households was $85,00 in 2019, only Indian and Filipino subgroups had median incomes above this while Burmese and Nepalese subgroups had median incomes of $44,400 and $55,000, respectively. Additionally, Southeast Asians are more likely to live in poverty compared to East Asians in the U. S. which is thought to be the result of both premigration and postmigration experiences, such as limited opportunities for education and work (Huang et al., 2012). Third, none of the variables in this study are specific to COVID-19 and therefore the role of COVID-19 in this model cannot be determined. Thus, the findings are interpreted in the context of the pandemic, but cannot be causally linked to COVID-19.

While the findings of this study informed the applicability of the FSM among AA families, further research is needed to examine other factors that may be uniquely influencing the relationship between economic stress and child outcomes among this population. For instance, the levels of acculturation (i.e., adapting to mainstream cultural norms) and enculturation (i.e., retaining the heritage cultural norms) may be important to include in the FSM model for AA families. Additionally, it is possible that economic stress impacts the development of parent depressive and anxiety symptoms differently; combining these symptoms into one construct may prevent the model from capturing unique relationships with other constructs of interest. Discrimination is another construct that should be added to future models, given the intersection between anti-Asian discrimination and disproportionate lay off rates for AA individuals during the COVID-19 pandemic, as well as the risk that discrimination poses to mental health (Hou et al., 2017). Collectivistic values, such as concern for face, preserving harmony, and prosocial motivation, may also be important to examine, as existing research indicates that collectivistic values influence how AA individuals manage various stressors (Chiu and Kosinski, 1995). Relatedly, future research should consider adapting existing parenting behavior measures to align with collectivistic values.

## Conclusion

Despite the limitations, the current study addresses a critical gap in the literature by examining the generalizability of the FSM to AA families. To start, it is important for clinicians to be aware of how economic stress uniquely impacts AA families so that appropriate interventions aimed at targeting adverse child outcomes can be developed, implemented, and disseminated throughout AA communities. Specifically, the results of this study demonstrate the importance of targeting treatment at the family level when working with children from families that are at risk for economic stress. Interventions focused on improving parent mental health and promoting positive parenting behaviors during times of economic hardship should be used to reduce or prevent child externalizing behaviors. By teaching parents concrete skills that emphasize positive parenting, clinicians can help foster positive development for youth exposed to economic stress.

Furthermore, this study offers a novel and optimistic contribution to the literature that highlights the resiliency of AA parents who are already demonstrating positive parenting behaviors in the face of economic stress. Therefore, clinicians should utilize a strengths-based and individualized approach when working with AA families. For example, for a parent who already utilizes positive reinforcement and proactive parenting techniques, the focus of treatment should be on increasing limit setting and engagement. Clinicians can also help parents identify adaptive ways of coping with economic stress and ways to discuss the topic in developmentally appropriate ways with their children. In a recent study, cognitive reframing skills (i.e., focusing on gratitude, developing positive meanings about the situation, etc.) were found to help individuals cope with financial stress and promoted family well-being (Kelley et al., 2023). Clinicians can also work collaboratively with AA parents to determine how to implement positive parenting behaviors in a way that aligns with the family’s cultural values.

Although this study was conducted during the COVID-19 pandemic, the clinical implications extend beyond the context of the pandemic as many AA families experience financial stressors every day. Using a prevention framework, addressing parent mental health and parenting behaviors among economically vulnerable families, even before signs of child problems are present, could have important protective effects on child development. Furthermore, the longitudinal findings of this study indicate that positive parenting behaviors can have lasting beneficial effects. Therefore, providing free or low-cost workshops on effective parenting strategies could offer protective effects for children not only in times of economic stress, but also for future stressors that families may face.

While these results highlighted specific family processes for clinicians to target when working with child externalizing problems, the direct link between economic stress and child internalizing problems must be noted. This finding is particularly concerning given that economically stressed families, especially racial/ethnic minority families, are more likely to experience barriers to mental health care such as waitlists, financial cost, lack of insurance, and lack of transportation (Ofonedu et al., 2017). Thus, the very children who are at higher risk for developing internalizing problems due to economic stress are the same children who face more barriers to treatment. One way of addressing this problem focuses on expanding access to mental health services for at-risk children and their families. This includes providing school-based mental health services, reducing cost, using telehealth interventions, and offering flexible treatment times to accommodate parents’ work schedules.

Lastly, policy changes are also needed at the federal, state, and local levels to address vulnerability to economic stress. Policy efforts directly aimed at alleviating economic stress among at-risk families, such as anti-poverty programs and free financial planning services, can help reduce social inequality and buffer the harmful effects of economic stress on parents and families. Furthermore, financial support systems that specifically recognize the diversity within AA communities are needed. This includes the development and expansion of language-accessible financial education materials, translation services, and outreach efforts conducted through community-based organizations that are already embedded in AA communities. These culturally responsive interventions are essential not only for reducing social and economic inequality among AA families, but also for mitigating the psychological and relational harm caused by financial strain. Overall, the results of this study provide critical insights into the needs of AA families experiencing economic stress as well as important implications for how clinicians, researchers, and policymakers can appropriately address these needs with the goal of fostering positive family functioning and youth development.

## Data Availability

The raw data supporting the conclusions of this article will be made available by the authors, without undue reservation.
